# A revised neuropsychological test battery, CoCoBattery-Plus, for the diagnosis of HIV-associated neurocognitive disorders in Japan

**DOI:** 10.1186/s12981-025-00835-4

**Published:** 2026-01-21

**Authors:** Aya Nakao, Kensuke Komatsu, Ai Takahashi-Nakazato, Aki Watanabe, Daisuke Tominaga, Shinichi Oka, Tatsuya Konishi, Kentaro Kawabe, Jun Yamanouchi

**Affiliations:** 1 Department of Hematology, Clinical Immunology, and Infectious Diseases, Ehime University Graduate School of Medicine, Ehime, Japan; 2 Faculty of Human Sciences, Wako University, Tokyo, Japan; 3 AIDS Clinical Center, National Center for Global Health and Medicine, Japan Institute for Health Security, Tokyo, Japan; 4 Tokyo Metropolitan Children’s Medical Center, Tokyo, Japan; 5 University of the Ryukyus, Okinawa, Japan; 6 National Sanatorium Tama Zenshoen, Tokyo, Japan; 7 Division of Blood Transfusion and Cellular Therapy, Ehime University Hospital, Ehime, Japan; 8 Department of Child Psychiatry, Ehime University Graduate School of Medicine, Ehime, Japan

**Keywords:** Screening, HIV-associated neurocognitive disorder, Neuropsychological test battery, Subjective impairment, CoCoBattery

## Abstract

To diagnose HIV-associated neurocognitive disorders (HAND), several neuropsychological test batteries have been used in various studies and countries. In Japan, the Co-developing Comprehensive Neuropsychological Test Battery (CoCoBattery) was developed during a nationwide study conducted between 2014 and 2016 (the J-HAND study) to explore the prevalence of HAND and has been widely used thereafter. It consists of 14 tests covering eight key cognitive domains: language, attention/working memory, executive function, learning, memory, information-processing speed, visuospatial construction, and motor skills. However, some cases have been difficult to classify in terms of HAND severity due to the lack of subjective impairment assessments in CoCoBattery. Therefore, we added cognitive screening questions to CoCoBattery (CoCoBattery-Plus) and compared the results among 103 HIV-positive individuals. Using the original battery, 10 cases were diagnosed with HIV-associated dementia (HAD), 13 with mild neurocognitive disorder (MND), 39 with asymptomatic neurocognitive impairment (ANI), and 41 with no HAND. In contrast, using the new battery, four individuals who were previously unaware of cognitive impairment reported subjective complaints in response to the questions, leading to diagnostic changes: one case from ANI to HAD and three from ANI to MND. The final diagnoses were 11 HAD, 16 MND, and 35 ANI, corresponding to a reclassification rate of 3.9%. Subjective complaints are a crucial component in determining the severity of HAND, and we anticipate that CoCoBattery-Plus will enable more accurate HAND diagnosis.

## Introduction

Human immunodeficiency virus (HIV)-associated neurocognitive disorders (HAND) encompass varying degrees of cognitive impairment and continue to affect the quality of life of people living with HIV (PLWH). Accurate assessment and classification of HAND remains essential in clinical practice, particularly because mild impairment can affect daily functioning and treatment adherence [1, 5].

HAND is classified into three severity categories according to the Frascati criteria [1]: asymptomatic neurocognitive impairment (ANI), HIV-associated mild neurocognitive disorder (MND), and HIV-associated dementia (HAD). Under these criteria, HAND is diagnosed when an individual scores at least one standard deviation below the normative mean in two or more assessed cognitive domains. Common manifestations of HAND include impairments in memory, attention, processing speed, motor coordination, and mood or affect (e.g., apathy or depression). Even mild neurocognitive decline may increase the risk of unemployment, social isolation, and other psychosocial challenges [19]. Therefore, mental health professionals working in HIV care must be able to recognize these impairments and provide appropriate support strategies. Note that functional decline is required for classifying MND and HAD, but is absent in ANI. Although functional impairment is not required to diagnose HAND itself, subjective cognitive complaints in clinical practice may suggest possible functional difficulties and help refine the severity classification, which is intended to refine HAND severity classification rather than duplicate the information obtained from objective neuropsychological testing.

HAND diagnosis and assessment typically follow the Frascati criteria, which recommend testing at least five distinct cognitive domains: language, attention/working memory, executive function, learning, memory, information-processing speed, visuospatial construction, and motor skills [1]. However, previous reports from Japan indicated that different facilities employed heterogeneous neuropsychological tests, many of which lacked comprehensive coverage of the recommended cognitive domains [30]. Moreover, many standard neuropsychological batteries require over an hour of testing, placing a significant burden on both patients and clinicians. Several brief HAND screening tools, such as the HIV Dementia Scale (HDS) and the International HIV Dementia Scale (IHDS), are available internationally; however, these instruments primarily detect more advanced impairments and provide limited coverage of the cognitive domains required under the Frascati criteria. In contrast, CoCoBattery-Plus was designed to offer broader domain coverage within a clinically feasible timeframe and to align with the Frascati-based neuropsychological assessment protocols. Some studies have reported associations between subjective cognitive complaints and objective impairments or everyday functioning in PLWH, although the findings remain inconsistent [7].

During the nationwide J-HAND study conducted between 2014 and 2016 to explore the prevalence of HAND [17, 20], a collaborative group of HIV specialists and clinical psychologists developed a standardized yet efficient Japanese neuropsychological test battery, named the Co-developing Comprehensive Neuropsychological Test Battery (CoCoBattery). This battery consists of 14 tests covering eight key cognitive domains: language, attention/working memory, executive function, learning, memory, information-processing speed, visuospatial construction, and motor skills [34].

Subjective complaints are a crucial component in diagnosing the severity of HAND. However, CoCoBattery did not include measures of subjective impairment, which made it difficult to determine HAND severity in some cases. In this study, we reassessed the applicability of CoCoBattery after 12 years and aimed to address the battery’s limitations.

## Materials and methods

First, we reviewed the development process of CoCoBattery to evaluate its completeness. Then, to incorporate subjective impairment assessments and strengthen CoCoBattery for obtaining more accurate diagnoses, including HAND severity, we selected three cognitive screening questions recommended by the European AIDS Clinical Society (EACS) and examined them in a separate cross-sectional investigation conducted at seven general hospitals in western Japan. These questions demonstrated moderate sensitivity (32%) but high specificity (80%) for detecting cognitive impairment in PLWH [24]. In this preliminary study, 103 PLWH suspected of having HAND were enrolled [21].

## Results

### Review of the development process of CoCoBattery

When CoCoBattery was developed, 30 studies utilizing neuropsychological evaluations for HAND in adults (≥ 20 years) were identified through PubMed using combinations of keywords, including “HIV-associated neurocognitive disorder” [5], “neurocognitive test,” and “neuropsychological test.” These 30 studies collectively employed 67 distinct neuropsychological tests, 27 of which had established standardized data for Japanese populations [30].

To narrow the pool of 67 tests to a final battery, the working group focused on four primary considerations. First, they examined whether each test had demonstrated clear utility in prior HAND research, as indicated by its frequency of use in the reviewed studies. Second, each test needed to have standardized Japanese normative data to enable valid score interpretation. Where Japanese normative data were unavailable (e.g., Trail Making Test and Grooved Pegboard), widely used international norms were applied, with age and, when available, education adjustments specified in those references. Third, because the goal was to minimize total administration time, each candidate test had to be relatively short and practical. Finally, feasibility in routine clinical settings was essential; the team ensured that the selected tests would not require extensive training, complex scoring procedures, or difficult-to-obtain materials.

Through this process, 14 tasks were selected that collectively cover eight cognitive domains, resulting in the development of CoCoBattery. CoCoBattery is a comprehensive neuropsychological test battery that evaluates eight cognitive domains using 14 standardized test items and can be completed in approximately 40 min, providing both convenience and efficiency (Table 1).

**Table 1:** CoCoBattery: test characteristics and cognitive domains

Cognitive domain	Neuropsychological test	Examples of additional optional tests
Processing speed	Digit Symbol Coding, Trail Making Test–A	–
Visuospatial construction	Rey–Osterrieth Complex Figure Test (Copy)	Block Design
Learning/ memory	Rey–Osterrieth Complex Figure Test (Immediate, Delayed) Story Recall (Immediate, Delayed)	Prospective Memory Tasks (Appointment, Belongings, Picture-Card)
Language	Verbal Fluency Test (Category, Letter)	–
Executive function	Trail Making Test–B	Stroop Color–Word Test Wisconsin Card Sorting Test (WCST) Similarities (WAIS)
Attention/ Working Memory	Digit Span (Forward and Backward)	Tapping Span Auditory Serial Addition Test PASAT (Paced Auditory Serial Addition Test)
Motor skills	Grooved Pegboard (Dominant and Non-dominant Hand)	–

### Characteristics of the neuropsychological tests in CoCoBattery

#### Symbol coding (WAIS-III)

As an established indicator of information-processing speed, Symbol Coding is widely used in neuropsychological assessments and was frequently employed in previous studies [13]. In addition, the stimulus materials and scoring procedures are internationally standardized and widely adopted. This task involves several abilities, including processing speed, visuomotor coordination, sustained attention, and motor persistence [32, 35]. Activation across the frontal lobe and the frontoparietal cortical network—specifically the bilateral inferior frontal sulcus, the left middle frontal gyrus, and the left posterior parietal lobe—has been associated with this task [33].

#### Rey complex figure test

This test primarily assesses visual memory, learning, visuospatial construction, executive function, and fine motor skills. While the entire cerebrum is involved, the frontal lobe contributes to drawing strategies and planning, whereas the parietal and occipital lobes are involved in spatial construction [8, 35]. Although the Rey provides broad coverage, it is a two-dimensional drawing task and therefore has inherent limitations in capturing three-dimensional aspects of visuospatial construction. We considered block-construction tasks such as Block Design [32], but standardized versions typically require approximately 15 min, which was incompatible with the < 40-minute time frame of the battery. Because the assembly strategy and stepwise organization observed during block tasks can provide clinically informative insights, we recommend adding a standardized block-construction task in clinical settings when a more detailed evaluation of visuospatial construction is warranted.

#### Story recall (the rivermead behavioural memory test, RBMT)

This task assesses auditory memory, learning, episodic memory, and sustained attention. It is associated with the left frontal, temporal, and parietal lobes, which are involved in processing verbal information [14]. Both the stimulus materials and scoring criteria are internationally standardized and widely used [35]. Immediate recall primarily reflects linguistic encoding and contextual understanding, with contributions from the left hippocampus and frontotemporal association cortices [6]. Delayed recall reflects the retention and consolidation of episodic memory, engaging distributed neocortical networks together with medial temporal structures. Moreover, Story Recall is a well-established measure, and the availability of four parallel versions makes it suitable for repeated testing [14]. Although the Wechsler Memory Scale—Revised (WMS-R) provides a comprehensive verbal memory assessment, its administration time exceeds the CoCoBattery’s 40-minute constraint and was therefore not included.

#### Verbal fluency test

This task assesses not only language abilities but also executive functions such as lexical retrieval and information manipulation. The CoCoBattery working group classified it under the Language domain. Letter fluency was assessed using the Japanese letters ‘Ka,’ in accordance with standard Japanese neuropsychological norms. The medial temporal lobe is primarily engaged in category fluency tasks, whereas the left dorsolateral prefrontal cortex and cingulate gyrus are involved in letter fluency tasks [3, 4]. This function is vulnerable to lesions in either hemisphere of the frontal lobe, with the most pronounced decline observed after left dorsolateral prefrontal lesions [27, 36]. Fluency deficits, however, are not specific to frontal injury alone and have also been reported following left parietal damage [27].

#### Trail making test A/B (TMT-A/B)

This test assesses processing speed, visuomotor coordination, sustained and selective attention, and visual search and scanning abilities. Both the stimulus materials and scoring procedures are internationally standardized and widely used [11]. TMT-B additionally requires attentional switching and executive functioning. Performance on this test is primarily associated with the left frontal lobe, including the left dorsolateral prefrontal cortex, primary motor cortex, cingulate gyrus, and left superior and middle temporal gyri [2, 10, 11]. Because executive function carries different interpretive weight compared with other domains, test selection for this domain prompted the most discussion among the working group. For interpretation, a disproportionately prolonged TMT-B relative to TMT-A (a B/A time ratio > 3.0) suggests impaired cognitive flexibility rather than generalized psychomotor slowing [29]. Although TMT-B alone does not comprehensively assess executive processes, we prioritized brief administration time and therefore did not add further executive measures to the core battery. The Stroop Color–Word Test, which was initially considered and is frequently used alongside TMT in prior studies, is recommended as an additional assessment when executive dysfunction is suspected [15].

#### Digit span (WAIS-III)

This test measures attention, working memory, and short-term memory and is associated with widespread cerebral activation. The stimulus materials and scoring procedures are internationally standardized and widely used [32]. Forward and backward spans are evaluated separately, with forward span primarily interpreted as a measure of attention and backward span as a measure of working memory. Backward digit span is more closely associated with activation in the left occipital lobe (visual areas) and left prefrontal cortex, which support central executive functions and visuospatial processing [31, 32]. Although Digit Span is an auditory task, the Tapping Span—which assesses visual working memory—was considered as an alternative [28]. The Letter-Number Sequencing task was also considered, but because of its difficulty, it was expected to cause participant fatigue [9]. Both were therefore added as optional supplemental tests.

#### Grooved pegboard test

This test requires participants to insert grooved pegs into matching slots using one hand (dominant and non-dominant), and the completion time is recorded [33]. It assesses fine motor skills (manual dexterity) and visuomotor coordination and is associated with activation of networks involving the primary motor cortex, premotor cortex (including the supplementary motor area), and superior parietal lobule [23].

#### Pre- and post-battery screening test

Brief screening instruments are administered before and after CoCoBattery to protect result validity and provide additional clinical information. Before CoCoBattery, an assessment of global cognitive function, such as the Mini-Mental State Examination (MMSE), is performed [26]. Pre-battery screening helps confirm that participants can meaningfully complete CoCoBattery and, if necessary, guides adjustments to pacing or the selection of alternative evaluations. After CoCoBattery, instruments such as the Profile of Mood States (POMS) and the Mini International Neuropsychiatric Interview (MINI) are administered to detect emotional or psychiatric factors that may affect test performance [16, 17]. These assessments help identify emotional states such as depression, anxiety, and fatigue that may influence cognitive scores and prompt referrals for mental health care. Although these screening measures were not part of the formal CoCoBattery protocol and were excluded from the HAND domain scoring, they were used to confirm test feasibility (MMSE) and aid clinical interpretation (POMS, MINI). In accordance with previous CoCoBattery studies, individuals with acute psychiatric illnesses, uncontrolled major depressive episodes, or non–HIV–related neurological disorders were excluded during routine clinical evaluations before neuropsychological testing. Although formal scales for fatigue or pain (e.g., VAS) were not administered, post-test assessments using the POMS and MINI ensured that severe mood or anxiety symptoms were not present and supported the appropriate cognitive performance interpretation. Because the Wide Range Achievement Test, Fourth Edition (WRAT4) is not standardized for the Japanese population, premorbid functioning was not assessed using the WRAT4.

### Administration order and considerations

An alternating sequence of verbal and motor tasks was designed to maintain participant engagement and ensure appropriate spacing between immediate and delayed memory tasks. The complete test sequence is presented in Table 2. This arrangement also minimizes fatigue or frustration that might occur when consecutive tasks of the same modality are administered. When administering CoCoBattery, clinicians are encouraged to document participant behavior—such as signs of weariness, inattention, or distress—and to consider these observations in the interpretive process. This qualitative information may offer insights that raw scores alone cannot provide and can guide decisions about whether additional testing is warranted. This alternating structure was originally adopted in the J-HAND study [17] to minimize order effects, which were not evaluated in the present study. The alternating verbal–motor task sequence also functioned as a natural filler interval between the immediate and delayed recall tasks, which is consistent with the procedure used in the original J-HAND study.

**Table 2:** Administration order and approximate duration

*A: Pre-Battery screening*		
0.	Mini–Mental State Examination (MMSE)	10 min
*B: CoCoBattery*		
1.	Digit Symbol Coding	5 min
2.	Rey–Osterrieth Complex Figure Test (Copy)	3 min
3.	Story Recall (Immediate)	1.5 min
4.	Rey–Osterrieth Complex Figure Test (Immediate Recall)	3 min
5.	Verbal Fluency Test (Category, Letter)	3 min
6.	Trail Making Test–A and –B	5 min
7.	Digit Span (Forward, Backward)	10 min
8.	Grooved Pegboard (Dominant and Non-dominant Hand)	5 min
9.	Story Recall (Delayed)	1.5 min
10.	Rey–Osterrieth Complex Figure Test (Delayed Recall)	3 min
*C: Post-Battery Psychological Assessment*		
11.	Profile of Mood States (POMS), etc.	5 min

### Scoring and interpretation

Standardized scores (z-scores or T-scores) were derived from Japanese age-adjusted norms when available, and from widely used international norms when Japanese references were lacking. Education adjustments were applied only when specified in the normative reference. In line with the Frascati criteria, scoring ≥ 1 SD below the normative mean in two or more cognitive domains indicates HAND, prompting further evaluation or clinical intervention. Accurate interpretation is essential, requiring clinicians to consider potential confounders such as psychiatric comorbidities, substance use history, and other medical conditions common among people living with HIV. The CoCoBattery-Plus consists exclusively of standardized neuropsychological tests for which Japanese normative data or validated international norms are available. Because this study’s purpose was to examine the effect of adding subjective screening items on HAND severity classification, formal psychometric validation of the combined battery (e.g., internal consistency, test–retest reliability, or construct validity) was not performed and should be addressed in future research. CoCoBattery subtests’ detailed descriptive statistics have been reported previously [16], and this study did not intend to reanalyze the data.

### Application of CoCoBattery-plus in a clinical study

Participants were evaluated using the same clinical procedures as in previous CoCoBattery studies, where exclusions (e.g., acute psychiatric illness and non-HIV-related neurological disorders) were already applied. This study did not include any new exclusion criteria. This study’s primary aim was to compare diagnostic classifications between individuals using the two test batteries; demographic and HIV-related clinical variables were not used as covariates in the analyses. Trained clinical psychologists familiar with the CoCoBattery protocol performed all assessments. The examiners were not blinded to the clinical information, as the study compared the diagnostic classifications within individuals.

Overall, CoCoBattery was well designed for screening HAND among PLWH. However, to evaluate HAND severity, subjective complaints are a critical component. Therefore, we added cognitive screening questions (Table 3) recommended by the EACS [] to CoCoBattery, creating the revised version named CoCoBattery-Plus (Tables 2 and 3), and applied it in a multicenter cross-sectional study [21]. In this study, HAND was diagnosed in 62 of 103 PLWH (60.2%) who were suspected of having HAND.

**Table 3:** Cognitive screening questions recommended by the European AIDS clinical society (EACS)

1. Do you experience frequent memory loss?
2. Do you feel that you are slower when reasoning, planning activities, or solving problems?
3. Do you have difficulties paying attention?

EACS, European AIDS Clinical Society

Using the original CoCoBattery, 10 cases were diagnosed with HAD, 13 with MND, 39 with ANI, and 41 with no HAND. In contrast, using CoCoBattery-Plus, four individuals who had not been aware of cognitive impairment reported subjective complaints in response to the questions, leading to diagnostic changes: one case from ANI to HAD and three cases from ANI to MND. The final diagnostic distribution was 11 HAD, 16 MND, and 35 ANI (Table 4). Functional status relevant to distinguishing between ANI, MND, and HAD was assessed clinically based on routine interviews and a medical records review by HIV specialists, consistent with procedures in previous CoCo battery studies. No additional structured functional assessments were performed during neuropsychological testing sessions.

**Table 4:** Severity of HAND diagnosed by cocobattery and cocobattery-Plus (*n* = 103)

Severity of HAND	CoCoBattery	CoCoBattery-Plus
HAD	10	11
MND	13	16
ANI	39	35
Others	41	41

ANI, asymptomatic neurocognitive impairment; CoCoBattery, Co-developing Comprehensive Neuropsychological Test Battery; HAD, HIV-associated dementia; HAND, HIV-associated neurocognitive disorders; MND, mild neurocognitive disorder.

## Discussion

We reviewed CoCoBattery and confirmed that it effectively balances comprehensiveness with clinical feasibility for diagnosing HAND by covering eight cognitive domains in approximately 40 min. Its use in the J-HAND study demonstrated that trained clinicians from multiple facilities could consistently implement the protocol, suggesting strong external validity and practicality [17, 20]. However, when PLWH did not recognize their cognitive impairment due to advanced disease stage or reported no subjective difficulties, the severity of HAND was sometimes misclassified, as seen in four cases in this study (Table 4). For this reason, the addition of cognitive screening questions is necessary to improve diagnostic accuracy. In the present study, four individuals who were reclassified after adding the screening questions initially reported no subjective cognitive difficulties. However, structured questions elicited concrete examples of everyday problems such as forgetting essential items during routine activities, difficulty in planning or organizing tasks, missing appointments, and trouble sustaining attention during reading. These previously unreported issues suggest functional concerns relevant to distinguishing ANI from MND and HAD. Because only four individuals experienced reclassification, and these shifts reflect diagnostic refinement within individuals rather than differences between groups, the shift rates’ statistical comparison was not conducted. Although such difficulties may not necessarily be solely attributable to HIV-associated neurocognitive impairment, early recognition of its functional impact allows clinicians to provide individualized support and timely interventions. Therefore, the CoCoBattery-Plus is expected to be widely adopted in future research and clinical practice. The subjective screening questions were used only to refine the severity classification and were interpreted with caution, given their limited and inconsistent relationship with objective performance.

The Frascati criteria specify that at least five cognitive domains must be evaluated for HAND diagnosis but impose no upper limit on the number assessed [1]. Accordingly, we prioritized maximal cognitive coverage within the practical constraint of an approximately 40-minute session, which we believe facilitates timely referral for targeted neuropsychological or psychosocial support. Furthermore, multidimensional tasks such as the Rey Complex Figure Test were included because they have robust Japanese normative data [14] and are reimbursable under the national health insurance system, supporting routine and sustainable use of CoCoBattery. Although visuospatial construction deficits are less frequently emphasized in HIV-related cognitive impairment, this domain remains a part of the Frascati criteria and contributes clinically meaningful information, particularly for differentiating broader cognitive profiles. Adding the three screening questions requires approximately five additional minutes, which we believe maintains the feasibility of CoCoBattery-Plus for routine clinical use.

However, several limitations remain. First, some participants may require supplemental testing. When the battery alone is insufficient to identify specific impairments, additional assessments may be necessary to capture the full clinical picture. For example, mood and depression can be evaluated using the Beck Depression Inventory (BDI)[18], and premorbid intellectual functioning can be assessed using the Japanese Adult Reading Test (JART)[22]. Additionally, the Iowa Gambling Task (IGT) may be useful for evaluating executive function and decision-making ability [25]. As mentioned above, because CoCoBattery prioritizes brevity and assesses executive function and visuospatial construction with only one task each, additional measures such as the Wisconsin Card Sorting Test (WCST)[12], Stroop Test [31], or tasks such as cube drawing, Block Design (WAIS) and Object Assembly may be warranted for a comprehensive assessment of deficits. Social cognition tests were not included because validated Japanese normative data are limited and because this domain was outside the original CoCoBattery design’s intended scope. When incorporating additional tests, however, it is essential to limit them to the minimum necessary to avoid overburdening participants. Second, only a subset of the tests used in the international HAND literature have established standardized Japanese normative data, highlighting the need for further adaptation and validation. Moreover, because the CoCoBattery and its normative references were developed primarily from Japanese samples, the CoCoBattery-Plus’s generalizability to other East Asian or non-Japanese populations remains uncertain, and cross-cultural validation, including normative data collection outside of Japan, is essential for broader implementation. Lastly, expanding normative data for older PLWH could enhance the diagnostic accuracy of CoCoBattery-Plus. As subjective complaints may be influenced by mood, these items should be interpreted with caution when determining HAND severity, and clinicians must consider the potential susceptibility to emotional states when integrating them into the diagnostic process. Previous studies have shown only limited and inconsistent associations between subjective complaints and objective cognitive impairment or functional outcomes in PLWH [7]. Moreover, most of these findings were derived from Western cohorts, and data from Japanese or East Asian populations remain extremely limited, underscoring the need for further local validation. Furthermore, mood symptoms, health-related anxiety, cultural factors such as stigma, and a tendency to minimize or underreport cognitive concerns may influence the accuracy of self-reported difficulties. These issues highlight the need for cautious interpretation of subjective complaints and future work examining how cultural contexts affect the use of self-reports in HAND assessment. Future studies should also examine how demographic and HIV-related clinical factors, such as CD4 count, disease duration, or ART status, may influence HAND severity classification when using the CoCoBattery-Plus.

Nationwide use of CoCoBattery-Plus will not only improve the assessment of neurocognitive deficits in PLWH but also contribute to a standardized approach for neurocognitive screening and integrated psychosocial support. Continued validation and refinement are anticipated, positioning CoCoBattery-Plus as a cornerstone of Japanese HIV neurocognitive research and clinical care. Future research should validate the CoCoBattery-Plus in older PLWH, individuals with lower educational backgrounds, and culturally diverse populations. Longitudinal studies are also required to determine whether the added subjective items improve the early detection of cognitive decline over time.

We anticipate that CoCoBattery-Plus is feasible for nationwide implementation in Japan and will enable more accurate HAND diagnosis.

## Data Availability

No datasets were generated or analysed during the current study.
